# A simple, fast and reproducible echocardiographic approach to grade left ventricular diastolic function

**DOI:** 10.1007/s10554-015-0832-6

**Published:** 2016-02-03

**Authors:** Bas M. van Dalen, Mihai Strachinaru, Julio van der Swaluw, Marcel L. Geleijnse

**Affiliations:** Department of Cardiology, The Thoraxcenter, Erasmus University Medical Center, ‘s-Gravendijkwal 230, Room Bd412, 3015 CE Rotterdam, The Netherlands; Department of Cardiology, Sint Franciscus Gasthuis, Rotterdam, The Netherlands

**Keywords:** Echocardiography, Diastolic function, Diastolic dysfunction

## Abstract

The American Society of Echocardiography and European Association of Echocardiography (ASE/EAE) have published an algorithm for the grading of diastolic function. However, the ability to use this algorithm effectively in daily clinical practice has not been investigated. We hypothesized that in some patients it may be difficult to grade diastolic dysfunction with this scheme, since there may be discrepancies in the assessed parameters. The aim of the current study was to test the feasibility of the ASE/EAE algorithm and to compare this with a new Thoraxcenter (TXC) algorithm. The ASE/EAE and TXC algorithms were applied to 200 patients. The ASE/EAE algorithm starts with assessment of diastolic myocardial wall velocities and left atrial (LA) volumes with subsequent assessment of E/A ratio, E-wave deceleration time and pulmonary venous flow. The TXC algorithm reverses these steps, uses LA dimension instead of volume and does not include a Valsalva manoeuvre and pulmonary venous flow. Due to inconsistencies between diastolic myocardial wall velocities and LA volumes and a not covered E/A ratio in the range of 1.5–2 it was not possible to classify 48 % of patients with the ASE/EAE algorithm, as opposed to only 10 % by the TXC algorithm. LA volume was always needed in the ASE/EAE algorithm. In only 64 % of patients LA size was necessary by the TXC algorithm. When LA volume would have been used instead of LA dimension, grading of LV diastolic function would have been different in only 2 % of patients without apparent improvement. Assessment of LA dimension was considerably faster than LA volume. The TXC algorithm to grade LV diastolic dysfunction was compared to the ASE/EAE algorithm simpler, faster, better reproducible and yields a higher diagnostic outcome.

## Introduction

**Fig. 1:**
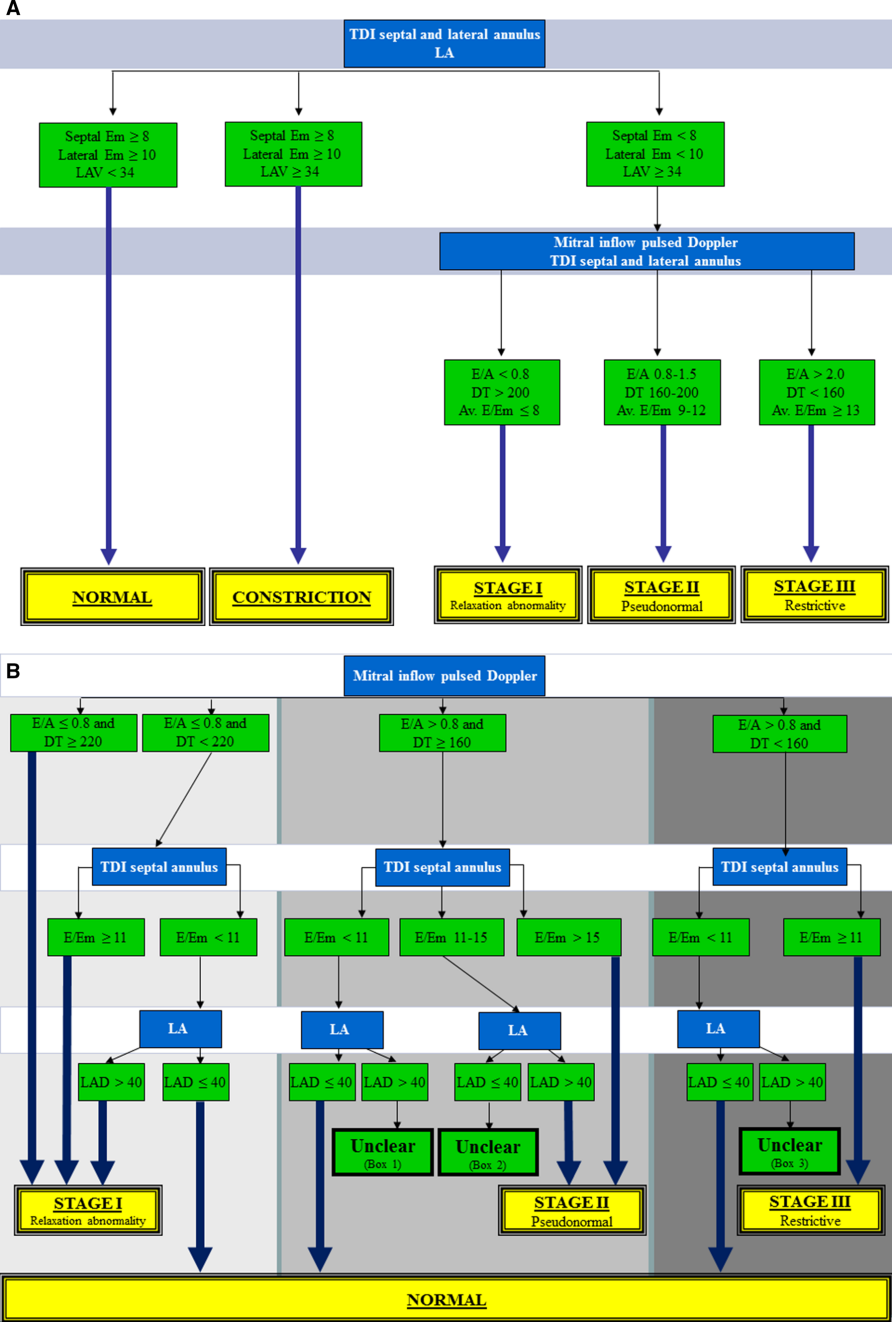
**a** Grading of left ventricular diastolic function according to the ASE/EAE algorithm, **b** Grading of left ventricular diastolic function according to TXC algorithm. *E* peak early filling velocity, *A* peak late filling velocity, *DT* E-velocity deceleration time, *Em* velocity of the mitral annulus early diastolic wave, *TDI* tissue Doppler imaging, *LA* left atrium, *LAD* left atrial dimension, *LAV* left atrial volume, *Av* averaged (from septal and lateral measurements)

Heart failure is a major public health problem in developed countries [1]. Left ventricular (LV) diastolic dysfunction is one of the important mechanisms responsible for symptoms in patients with heart failure, irrespective of the presence or severity of systolic LV dysfunction [2]. It has been well established that diastolic dysfunction and filling pressures can be assessed by two-dimensional and Doppler echocardiography [3, 4]. The American Society of Echocardiography and European Association of Echocardiography (ASE/EAE) have published a guideline for the echocardiographic assessment of diastolic function in various clinical conditions [5]. This ASE/EAE guideline contains a practical algorithm for grading diastolic dysfunction (Fig. 1a). The ASE/EAE authors claimed that this algorithm was an important predictor of all-cause mortality in an earlier large cross-sectional survey [6]. However, the ability to use this algorithm effectively in daily clinical practice has not been investigated. We hypothesized that in some patients it may be difficult to grade diastolic dysfunction with this scheme, since there may be discrepancies in the assessed parameters. Therefore, the aim of this study was to test the feasibility of the ASE/EAE algorithm and to compare this with a newly proposed Thoraxcenter (TXC) algorithm (Fig. 1b).

## Methods

### Study participants

The study population consisted of 200 consecutive patients (mean age 52 ± 15 year, 49 % female) referred for echocardiography in both a tertiary referral center (n = 85, Erasmus University Medical Center, Rotterdam, The Netherlands) and a smaller non-academical general hospital (n = 115, Sint Franciscus Gasthuis, Rotterdam, The Netherlands). Assessment of LV diastolic function had to be part of the echocardiography protocol and patients had to be in sinus rhythm. Athletes (international or national level of participation for at least 2 years) were excluded, as well as patients with hypertrophic cardiomyopathy, more than mild valvular disease, and a history of cardiac surgery.

In order to obtain a cut-off value for the ratio of peak early filling velocity (E) over mitral annulus early diastolic wave velocity (Em), 100 healthy control subjects (mean age 46 ± 14 year, female 49 %) in sinus rhythm, without hypertension, diabetes, or regular use of medication for cardiovascular disease, and with normal left atrial dimensions, LV dimensions, and LV ejection fraction were studied. Control subjects were recruited from our department (personnel) or were family members or friends.

The institutional review board approved the study.

### Echocardiography

Two-dimensional grayscale harmonic images were obtained in the left lateral decubitus position using a commercially available ultrasound system (iE33, Philips, Best, The Netherlands), equipped with a broadband (1–5 MHz) S5-1 transducer (frequency transmitted 1.7 MHz, received 3.4 MHz). All echocardiographic measurements were averaged from three heartbeats. Left atrial (LA) dimension was measured as the anterior-posterior diameter in an end-systolic parasternal image. LA volume was calculated using the biplane area-length formula and indexed for body surface area [7]. From the mitral-inflow pattern, E and peak late (A) filling velocities, E/A ratio, and E-velocity deceleration time (DT) were measured. Tissue Doppler imaging was applied by placing the sample volume at the side of the medial (septal Em) and lateral annulus (lateral Em) in an apical 4-chamber view [8]. For the ASE/EAE algorithm both septal and lateral Em were needed, whereas for the TXC algorithm only septal Em was mandatory. Gain and filter settings were adjusted as needed to eliminate background noises and to allow for a clear tissue signal. To acquire the highest tissue velocities, the angle between the Doppler beam and the longitudinal motion of the investigated structure was adjusted to a minimal level. Em was recorded end-expiratory at a sweep speed of 100 mm/s.

### Grading LV diastolic dysfunction

Two algorithms were used to grade diastolic dysfunction. The ASE/EAE algorithm was based on the scheme published in 2009 [5]:

- When septal Em was ≥8 cm/s, lateral Em ≥10 cm/s and/or LA volume <34 ml/m^2^ diastolic function was graded as normal.
- Since athletes were excluded, septal Em ≥8 cm/s, lateral Em ≥10 cm/s and/or LA volume ≥34 ml/m^2^ suggested constriction, although other clinical variables should be considered as well in that case.
- When septal Em was <8 cm/s, lateral Em <10 cm/s and/or LA volume ≥34 ml/m^2^ diastolic function was graded abnormal.

When it was not possible to grade diastolic dysfunction due to discrepancies in the assessed parameters, the exact reason was registered.

The newly proposed TXC algorithm was primarily based on the same study by Redfield et al. [6] that was used as the basis of the ASE/EAE algorithm. However, it starts with assessment of the E/A ratio and DT. Further subdivision was based on the E/Em ratio (using Em septal) and when necessary on LA dimension (rather than volume). E/Em ratio in the 100 healthy control subjects was 7.2 ± 1.9, leading to a cut-off value of 11 (mean ± 2SD).

- When the mitral E/A ratio was ≤0.8 and DT ≥220 ms diastolic function was graded as relaxation abnormality.
- When E/A ratio was ≤0.8 but DT was relatively short (<220 ms) for relaxation abnormality, E/Em and LA dimension were used to differentiate between normal diastolic function (E/Em <11 and LA ≤40 mm), and relaxation abnormality (E/Em ≥11 or E/Em <11 but LA >40 mm).
- When E/A ratio was >0.8 and DT ≥160 ms, again E/Em and LA dimension were used to differentiate between normal diastolic function (E/Em <11 and LA ≤40 mm), and pseudonormal diastole (E/Em 11–15 and LA >40 mm or E/Em >15).
- A short DT (<160 ms) suggested restrictive filling. However, in healthy adolescents and young adults, there may be a marked contribution of active LV relaxation to LV filling, resulting in a short DT that resembles a restrictive LV filling pattern. Yet, in these subjects E/Em was supposed to be <11 and LA dimension ≤40 mm.

In 50 randomly selected subjects the time needed to (offline) measure LA dimension and volume were assessed.

### Statistical analysis

Continuous variables are presented as mean ± SD and compared using Student’s t test. In the 50 randomly selected subjects in whom the time needed to measure LA dimension and volume were assessed, reproducibility of measurements were tested. Measurement variability was calculated as the mean per cent error, defined as the absolute difference between the two sets of measurements, divided by the mean of the measurements.

## Results

### Characteristics of the study population

In Table 1, the clinical and echocardiographic characteristics of the study population are shown. In 96 (48 %) patients it was possible to grade LV diastolic function by both algorithms. In only 2 out of these 96 patients there was a discrepant classification of LV diastolic function. According to clinical parameters such as age and final diagnosis, the newly proposed algorithm seemed to be correct in one patient (patient number 1, Table 2), while this is less obvious in the other patient, although both may be disputed. In the remaining 94 patients there was agreement with respect to classification of LV diastolic function by both algorithms. Normal diastole, relaxation abnormality, pseudonormal diastole and restrictive diastole in these latter 94 patients were found in 60, 19, 16, and 5 %, respectively.

**Table 1:** Clinical and echocardiographic characteristics of the study population

	Patients (n = 200)	Controls (n = 100)
Age (year)	52 ± 15	46 ± 14
Male, n (%)	101 (51)	51 (51)
Body surface area (m^2^)	1.9 ± 0.2	1.9 ± 0.2
Heart rate, beats/min	74 ± 11	72 ± 15
Systolic blood pressure (mmHg)	126 ± 18	122 ± 15
Diastolic blood pressure (mmHg)	77 ± 9	71 ± 10
Indication echocardiography, n (%)		
* First echocardiogram*	68 (34)	
Chest pain	11 (6)	
Dyspnea	24 (12)	
Paroxysmal atrial fibrillation	8 (4)	
Cardiac murmur	8 (4)	
Post myocardial infarction	13 (7)	
Syncope/sudden cardiac death	4 (2)	
* Follow-up*	132 (66)	
Post myocardial infarction	31 (15)	
Heart failure	39 (19)	
Valve disease	27 (14)	
Miscellaneous	35 (17)	
Diastolic echocardiographic characteristics		
Left atrial dimension (mm)	41 ± 6	30 ± 5
Normalized left atrial volume (ml/m^2^)	31.3 ± 10.4	23.1 ± 5.4
E-wave velocity (cm/s)	79 ± 20	66 ± 16
A-wave velocity (cm/s)	72 ± 18	52 ± 16
E/A ratio	1.1 ± 0.5	1.2 ± 0.5
E-velocity deceleration time, ms	203 ± 50	180 ± 36
Em septal (cm/s)	8.1 ± 2.7	9.6 ± 2.6
E/Em ratio	9.8 ± 4.6	7.2 ± 1.9

Values represent mean ± standard deviation. E-wave velocity = peak early phase filling velocity, A-wave velocity = peak late filling velocity, *Em* peak early diastolic wave velocity

**Table 2:** Patients with discrepant classification of left ventricular diastolic function by both algorithms

Number	LV diastolic function		Age (year)	Sex	Indication echocardiography	Clinical diagnosis	E/A ratio	E-velocity deceleration time (ms)	Em septal (cm/s)	E/Em ratio	Left atrial dimension (mm)	Normalized left atrial volume (ml/m^2^)
	ASE/EAE	TXC										
1	Normal	Pseudonormal	73	Female	Follow-up mild aortic regurgitation	HFPEF	1.9	181	9.4	11.3	42	33
2	Relaxation abnormality	Normal	69	Male	Chest pain	4 year post MI, good systolic LVF	0.7	199	5.8	6.9	36	35

*E/A ratio* ratio of peak early over peak late filling, velocity, *Em* peak early diastolic wave velocity, *HFPEF* heart failure with preserved ejection fraction, *MI* myocardial infarction, *LVF* left ventricular function, *ASE/EAE* American Society of Echocardiography and European Association of Echocardiography algorithm, *TXC* thoraxcenter algorithm

### Feasibility of both algorithms to grade diastolic dysfunction

It was not possible to grade LV diastolic function in 48 % of patients by the ASE/EAE algorithm. In contrast, only 10 % of the patients were not classified by the newly proposed TXC algorithm (*P* < 0.001). The reasons for failure to qualify LV diastolic function are shown in Table 3. When there was failure to grade LV diastolic function by the ASE/EAE algorithm, moderate to severe LV diastolic function (according to the TXC algorithm) was relatively abundant: normal diastole, relaxation abnormality, pseudonormal diastole and restrictive diastole were seen in 32, 26, 32, and 10 % of patients, respectively.

**Table 3:** Reasons for failure to classify left ventricular diastolic function

ASE/EAE algorithm, n (%)	
Normal Em but increased left atrial volume*	16 (8)
Decreased Em but normal left atrial volume	47 (24)
Decreased Em and increased left atrial volume but E/A ratio 1.5–2.0	7 (4)
Decreased Em and increased left atrial volume but discrepant E/A ratio and DT	27 (14)
Total	97 (48)
TXC algorithm, n (%)	
Normal E/A, DT and E/Em but increased left atrial dimension (Unclear Box 1**)	12 (6)
Normal E/A, DT and left atrial dimension but E/Em in “gray zone” (Unclear Box 2**)	5 (3)
Normal E/A and E/Em but short DT and increased left atrial dimension (Unclear Box 3**)	2 (1)
Total	19 (10)

*E/A ratio* ratio of peak early over peak late filling velocity, *DT* E-velocity deceleration time, *Em* peak early diastolic wave velocity, *DT* E-velocity deceleration time. *ASE/EAE* American and European Associations of Echocardiography, *TXC* thoraxcenter

* No constriction, ** According to Fig. 1b

Feasibility of both algorithms was also tested for the control group. It was not possible to grade LV diastolic function in 18 % of the controls by the ASE/EAE algorithm, mainly due to discrepancy between Em and LA volume (normal Em but increased LA volume in 4 % and decreased Em but normal LA volume in 10 %). Classification by the TXC algorithm was not possible in 4 % of the controls (2 in “Unclear Box 1”, 1 in “Unclear Box 2”, 1 in “Unclear Box 3”).

### Use of LA dimension versus LA volume

In the total group of patients there was discrepancy in 11 patients (6 %) with respect to the cut-off values of LA dimension (40 mm) and volume (34 ml/m^2^) used in the different algorithms. In 3 patients LA volume was ≥34 ml/m^2^ whereas LA dimension was <40 mm. On the other hand, in 8 patients LA dimension was ≥40 mm whereas LA volume was <34 ml/m^2^.

LA volume was per protocol always needed to qualify LV diastolic function in the ASE/EAE algorithm. In 128 patients (64 %) LA size was necessary to classify LV diastolic function by the TXC algorithm. When LA volume (cut-off value 34 ml/m^2^) would have been used instead of LA dimension (cut-off value 40 mm), grading of LV diastolic function by the TXC algorithm would have been different in only 3 patients (2 %) (Table 4). In two patients (patient number 1 and 3, Table 4), classification of LV diastolic function changed from unclear to normal, which in both patients may be correct. In the other patient (patient number 2, Table 4), use of LA dimension led to the seemingly correct diagnosis of pseudonormal LV diastolic function.

**Table 4:** Patients with different classification of left ventricular diastolic function when left atrial volume (≥34 ml/m^2^) was used instead of left atrial dimension (≥40 mm)

Number	LV diastolic function		Age (year)	Sex	Indication echocardiography	Clinical diagnosis	E/A ratio	E-velocity deceleration time (ms)	Em septal (cm/s)	E/Em ratio	Left atrial dimension (mm)	Normalized left atrial volume (ml/m^2^)
	LA dimension	LA volume										
1	Unclear (box 1*)	Normal	26	Male	Chest pain	Normal	1.0	169	9.7	7.6	45	28
2	Pseudonormal	Unclear (box 2*)	57	Male	Follow-up heart failure	DCM	0.9	170	4.5	11.6	47	32
3	Unclear (box 1*)	Normal	69	Male	Chest pain	Normal	0.9	216	7.7	9.3	43	29

*E/A ratio* ratio of peak early over peak late filling velocity, *Em* peak early diastolic wave velocity, *DCM* dilated cardiomyopathy. * According to Fig. 1b

Finally, assessment of LA dimension (6 ± 4 s) was considerably faster as compared to assessment of LA volume (40 ± 12 s, *P* < 0.001).

### Reproducibility

There was agreement between both observers in all subjects with respect to grading of LV diastolic function, irrespective of the algorithm used. The intra- and inter-observer variability of E/A ratio, DT, Em and E/Em were 4.8 ± 4.2 and 5.0 ± 4.4 %, 7.8 ± 5.2 and 8.4 ± 4.5 %, 5.0 ± 5.1 and 5.1 ± 4.4 %, and 5.9 ± 5.3 and 6.1 ± 4.9 %, respectively. Reproduciblity of LA dimension was better as compared to LA volume: intra- and inter-observer variability 4.8. ± 4.0 and 5.8 ± 4.2 % versus 8.8 ± 6.3 and 9.1 ± 5.9 %, respectively.

## Discussion

The most important conclusion of the current study is that in daily practice it is not possible to feasibly use the algorithm endorsed by ASE/EAE for grading LV diastolic function. On the other hand, the proposed TXC algorithm did allow assessment of LV diastolic function in 90 % of consecutive patients in sinus rhythm in an efficient manner.

A gold standard of LV diastolic function is lacking in the current study. However, the ASE/EAE algorithm also had never been validated against an invasive evaluation of LV diastolic function, although it is based on numerous studies that did use invasive standards. Nevertheless, both algorithms are based on these same landmark studies. Importantly, it should be noted that in the 48 % of patients with possible grading of diastolic function with both algorithms, no essential differences were found. Therefore, it seems unlikely that the newly proposed TXC algorithm would have different prognostic power compared to the ASE/EAE algorithm.

### Background of the ASE/EAE algorithm

The ASE end EAE have put commendable efforts in the publication in 2009 of a guideline for the echocardiographic assessment of diastolic function. It is an impressive document providing direction in this difficult aspect of echocardiography. Although it should be noted that the algorithm for qualification of diastolic function published in this guideline is supposed to be used in harmony with other potentially relevant parameters, the algorithm on itself does include several problems and inefficiencies.

Discrepancy between parameters (e.g. Em and LA volume or E/A ratio and DT) was the most important reason for failure to classify LV diastolic function. Also, an E/A ratio in the range of 1.5–2.0 is not covered in the algorithm, leading to unfeasibility to qualify some patients. In more recent studies, E/A ratio >1.5 was used as an indicator of stage III diastolic dysfunction [9, 10].

Septal and lateral Em and LA volume direct the primary differentiation between normal and abnormal LV diastolic function in the ASE/EAE algorithm to qualify LV diastolic function. The scientific background of the decision to create the algorithm in this manner was not fully elucidated in the paper in which the ASE/EAE algorithm was presented [5]. In the paper it was stated that the algorithm was based on findings of a large cross-sectional survey by Redfield et al. [6]. In this survey a combination of data from mitral inflow (E/A ratio and DT), tissue Doppler imaging (E/Em ratio) and pulmonary venous flow was used to qualify LV diastolic function. LV diastolic function was categorized as: *normal*; *mild* dysfunction, defined as impaired relaxation without evidence of increased filling pressures; *moderate* dysfunction, defined as impaired relaxation associated with moderate elevation of filling pressures or pseudonormal filling; and *severe* dysfunction, defined as advanced reduction in compliance or (reversible or fixed) restrictive filling. Redfield et al. based this classification on earlier publications by Nishimura [11] and Ommen et al. [12]. The study by Nishimura was a review from 1997, focusing on mitral inflow velocity curve patterns. Ommen et al. found the septal E/Em ratio to be the single best parameter for predicting mean LV diastolic pressure. However, from these studies, there seems to be no solid evidence in favour of using septal *and* lateral Em and in particular LA volume for the primary differentiation between normal and abnormal LV diastolic function. In fact, in 63 out of 200 patients in our study (see Table 3) there was a discrepancy between Em and LA volume, making it impossible to qualify LV diastolic function with this algorithm.

In the ASE/EAE algorithm, a cut-off value of 34 ml/m^2^ was chosen to differentiate between normal and abnormal LV diastolic function. The decision to choose this cut-off value was supported in the ASE/EAE paper by a reference to a review by Abhayaratna et al. [13]. However, the only studies identified by Abhayaratna et al. that found a LA volume of 34 ml/m^2^ to be the discriminatory threshold, were one case control study of atrial fibrillation in hypertrophic cardiomyopathy patients [14] and one study of subjects without a history of congenital heart disease, treatment with pacemaker implantation, valvular surgery, or cardiac transplantation, undergoing general medical consultation [15]. In the other 12 studies identified in this review cut-off values for LA volume ranging from 27 to 68 ml/m^2^ were found, depending on the study population and the chosen endpoints. Furthermore, in the recommendation paper by Lang et al. [7], a LA volume >29 ml/m^2^ was already considered abnormal, although this cut-off value has recently been adjusted to >34 ml/m^2^ [16].

### Background of the newly proposed TXC algorithm

For optimal application in daily clinical practice, any algorithm for qualification of LV diastolic function should be simple, fast and reproducible. The ASE/EAE scheme includes diastolic parameters that are more difficult to measure (less feasible) such as pulmonary venous flow. There is currently no evidence that assessment of these parameters is clinically relevant in a sense that they have independent incremental value over the more robust parameters for the assessment of LV filling pressures or overt heart failure. Therefore, in order to be as simple, fast and reproducible as possible, the newly proposed TXC algorithm did not include these LV diastolic function parameters.

Even more in-line with the aforementioned study by Redfield et al. [6], our algorithm starts with assessment of E/A ratio and DT. Since DT is normally between 160 and 220 ms [17] we used 220 ms as a cut-off value, instead of the 200 ms used in the ASE/EAE algorithm.

The E/Em ratio is known to correlate well with LV filling pressures [8]. Although either side of the mitral annulus can be used, septal E/Em has been shown to provide better diagnostic utility [18, 19], most likely because it is easier to align the tissue Doppler beam with the septal wall. For reasons of efficiency we decided therefore to use only the septal E/Em ratio. In two landmark papers in the field of E/Em ratio assessment [8, 12], different cut-off values for abnormal E/Em ratio have been reported. Ommen et al. [12] concluded that a septal E/Em ratio <8 suggests normal LV filling pressure, whereas >15 was highly specific for elevated LA pressure. Even though Nagueh et al. [8] found lateral Em to be slightly higher than septal Em, a lateral E/Em ratio >10 was already associated with increased LA pressure. Therefore, we have decided to define a normal value for E/Em ratio for our own department. Since E/Em was 7.2 ± 1.9 in healthy control subjects, a cut-off value of 11 (mean ± 2SD) was chosen.

Increased LA size is associated with adverse cardiovascular outcomes [20] since it is a marker of increased LA pressure over time [21]. A large volume of prior clinical and research work used the two-dimensional derived antero-posterior linear LA dimension obtained from the parasternal long-axis view, making this the standard for linear LA measurement [7]. Evaluation of the LA in the antero-posterior dimension assumes that a consistent relationship is maintained between the antero-posterior dimension and all other LA dimensions as the atrium enlarges, which is sometimes not the case [7, 22]. Expansion of the LA in the antero-posterior dimension may for example be constrained by the thoracic cavity between the sternum and the spine. Therefore, it has been advocated to use 2D (or even 3D) volumes rather than the antero-posterior dimension although data that show the superiority of LA volumes are rather sparse [23–30]. Nevertheless, in the present study we found that use of LA volume instead of LA dimension in the newly proposed TXC algorithm, would lead to a different classification of LV diastolic function in only 2 % of patients, without any evidence that it improves the correct classification of diastolic function. Since measurement of LA dimension was considerably faster and better reproducible as compared to LA volume measurement, we have chosen to still use LA dimension in the routine application of grading of diastolic function. A Framingham Heart Study cohort of 1099 subjects between the ages of 20 and 45 years old who were not obese, were of average height and were without cardiovascular disease, identified an anteroposterior LA dimension of 27–40 mm as the normal range [31]. Therefore, we have chosen to use an anteroposterior LA dimension of 40 mm as a cut-off value.

### Limitations

Validation of the new TXC algorithm against clinical outcome would be ideal. Yet, this was beyond the scope of the current paper but may be investigated in future studies. In order to represent daily clinical practice and to optimize feasibility of the new TXC scheme, further subdivision of abnormal diastolic function was only based on E/A ratio, DT, E/Em and LA dimension. In other words, although potentially helpful when there is discrepancy between different parameters, the relatively less used parameters “time difference between the pulmonary venous flow atrial reversal velocity waveform and mitral A-wave duration (Ar–A)” and “change of the E/A ratio with Valsalva maneuver (Val ΔE/A)” were not used. In future studies the incremental values of these variables should be shown before routine application may be advised. Also, when evaluating LV diastolic function, one may want to consider other echocardiographic variables such as the extent of LV hypertrophy, ejection fraction, ischemic wall motion abnormalities, and pulmonary pressure estimates. However, again, to optimize feasibility these parameters were not incorporated in the TXC algorithm, but of course each clinician should be free to use such variables as well when deemed necessary.

## Conclusion

Assessment of LV diastolic function is an essential part of most echocardiograms, in particular when heart failure is suspected. The newly proposed TXC algorithm to grade LV diastolic dysfunction is compared to the ASE/EAE algorithm simpler, faster, better reproducible and yields a higher diagnostic outcome. Simpler because use of LA size was less needed (64 vs. 100 %) and when needed a dimension rather than a volume was measured, only the septal mitral annular velocity was measured and less useful parameters such as pulmonary venous flow and use of a Valsalva maneuver were not included in the algorithm. It is faster because of the aforementioned arguments and for example the diagnosis of LV relaxation abnormality requires only 2 measures (E/A ratio and DT) rather than 5 measures (E/A ratio, DT, septal Em, lateral Em and LA volume) in the ASE/EAE algorithm. It is better reproducible because the intra- and inter-observer variability of the LA dimensions was lower compared to the LA volume and less parameters are involved. Finally, it yields better feasibility because a straight forward diastolic grade was defined in 90 % rather than 52 % of patients.

## Abbreviations

LV: Left ventricle (or ventricular)
LA: Left atrium (or atrial)
E: Peak early filling velocity
A: Peak late filling velocity
DT: Deceleration time of E-wave
Em: Velocity of the mitral annulus early diastolic wave
TXC: Thoraxcenter
ASE/EAE: American Society of Echocardiography and European Association of Echocardiography
